# A New Family of Lysozyme Inhibitors Contributing to Lysozyme Tolerance in Gram-Negative Bacteria

**DOI:** 10.1371/journal.ppat.1000019

**Published:** 2008-03-07

**Authors:** Lien Callewaert, Abram Aertsen, Daphne Deckers, Kristof G. A. Vanoirbeek, Lise Vanderkelen, Joris M. Van Herreweghe, Barbara Masschalck, Dorothy Nakimbugwe, Johan Robben, Chris W. Michiels

**Affiliations:** 1 Laboratory of Food Microbiology, Department of Microbial and Molecular Systems (M2S), Katholieke Universiteit Leuven, Leuven, Belgium; 2 Biomedical Research Institute (BIOMED), Hasselt University and Transnationale Universiteit Limburg, School of Life Sciences, Diepenbeek, Belgium; Massachusetts General Hospital, United States of America

## Abstract

Lysozymes are ancient and important components of the innate immune system of animals that hydrolyze peptidoglycan, the major bacterial cell wall polymer. Bacteria engaging in commensal or pathogenic interactions with an animal host have evolved various strategies to evade this bactericidal enzyme, one recently proposed strategy being the production of lysozyme inhibitors. We here report the discovery of a novel family of bacterial lysozyme inhibitors with widespread homologs in gram-negative bacteria. First, a lysozyme inhibitor was isolated by affinity chromatography from a periplasmic extract of *Salmonella* Enteritidis, identified by mass spectrometry and correspondingly designated as PliC (*p*eriplasmic *l*ysozyme *i*nhibitor of *c*-type lysozyme). A *pliC* knock-out mutant no longer produced lysozyme inhibitory activity and showed increased lysozyme sensitivity in the presence of the outer membrane permeabilizing protein lactoferrin. PliC lacks similarity with the previously described *Escherichia coli* lysozyme inhibitor Ivy, but is related to a group of proteins with a common conserved COG3895 domain, some of them predicted to be lipoproteins. No function has yet been assigned to these proteins, although they are widely spread among the Proteobacteria. We demonstrate that at least two representatives of this group, MliC (*m*embrane bound *l*ysozyme *i*nhibitor of *c*-type lysozyme) of *E. coli* and *Pseudomonas aeruginosa*, also possess lysozyme inhibitory activity and confer increased lysozyme tolerance upon expression in *E. coli*. Interestingly, *mliC* of *Salmonella* Typhi was picked up earlier in a screen for genes induced during residence in macrophages, and knockout of *mliC* was shown to reduce macrophage survival of *S.* Typhi. Based on these observations, we suggest that the COG3895 domain is a common feature of a novel and widespread family of bacterial lysozyme inhibitors in gram-negative bacteria that may function as colonization or virulence factors in bacteria interacting with an animal host.

## Author Summary

Lysozyme is an ancient bactericidal enzyme that is part of the antibacterial defense system of vertebrate and invertebrate animals. Bacteria colonizing or infecting an animal host have developed various ways to overcome lysozyme action, a recently proposed mechanism being the production of lysozyme inhibitors. However, the only high affinity bacterial lysozyme inhibitor known thus far is produced only in few bacteria, and this raised questions about their wider relevance in bacteria–host interactions. We here report the discovery of a novel and distinct family of bacterial lysozyme inhibitors that is widely distributed among the Proteobacteria, including several major pathogens. The family comprises periplasmic as well as membrane-bound inhibitors, and both types contribute to lysozyme tolerance of bacterial cells, as we experimentally demonstrate for the periplasmic inhibitor from *Salmonella* Typhimurium and the membrane-bound inhibitors from *Escherichia coli* and *Pseudomonas aeruginosa*. Interestingly, a gene encoding one of the newly identified inhibitors has been previously found to promote macrophage survival of *Salmonella* Typhi. The widespread occurrence of lysozyme inhibitors in bacteria is likely to reflect their functional importance in a wide range of bacteria–host interactions. As such, they are also attractive novel targets for antibacterial drug development.

## Introduction

Lysozymes (EC 3.2.1.17) hydrolyse the β-(1,4) glycosidic bond between *N*-acetylmuramic acid and *N*-acetylglucosamine in peptidoglycan, the major cell wall polymer in the Bacteria. Peptidoglycan forms a network that surrounds the entire bacterial cell, and its hydrolysis by lysozyme renders bacteria sensitive to lysis driven by turgor pressure. Lysozymes are implicated in defensive and offensive bactericidal systems in a wide range of taxonomically diverse organisms including fungi, protozoa, plants, invertebrate and vertebrate animals and even bacteriophages, indicating their evolutionary success as bactericidal tools. Most gram-negative bacteria are not susceptible to the action of lysozyme alone because their outer membrane prevents access of the enzyme to the peptidoglycan layer. However, this barrier has been overcome in the innate immune systems of animals by the production of accessory antibacterial proteins which permeabilize the outer membrane, such as lactoferrin. In addition, some natural lysozymes as well as chemically or genetically modified hen egg white lysozyme (HEWL) have been reported to be active against gram-negative bacteria even in the absence of such permeabilizers [1]–[4].

In view of the widespread occurrence and effectiveness of lysozymes as antibacterial agents, it is not surprising that bacteria have in turn evolved mechanisms to evade or subvert this threat. A bacterial lysozyme resistance mechanism that has been known for long is peptidoglycan modification. Examples are the de-N-acetylation of N-acetylglucosamine in *Bacillus subtilis* vegetative cells [5], and O-acetylation of the C-6 hydroxyl group of *N*-acetylglucosamine residues in *Staphylococcus aureus* and several other bacteria [6]. In *S. aureus*, this modification is carried out by a peptidoglycan-specific O-acetyltransferase encoded by *oatA*, and is believed to contribute greatly to the persistence of pathogenic *S. aureus* strains on the skin and mucosal surfaces [7]. A different bacterial strategy to evade the bactericidal action of lysozyme that has more recently emerged is the production of lysozyme inhibitors. In group A streptococci, a protein first identified as an inhibitor of the complement system and therefore designated as SIC (*s*treptococcal *i*nhibitor of *c*omplement), was later also shown to inhibit lysozyme [8]. However, since SIC does not have a very high affinity for lysozyme (dissociation constant K_d_  =  85.4 µM), and also binds to and inhibits several other components of the innate immune system such as secretory leukocyte proteinase inhibitor and β-defensins at higher affinity [8],[9], it can not be considered as a highly specific lysozyme inhibitor. A different lysozyme inhibitor, showing high affinity (K_d_  =  1 nM), was inadvertently identified during a systematic study of orphan gene products in *Escherichia coli* [10]. The product of *ykfE* was shown to strongly bind to and inhibit c-type lysozymes, which include HEWL and human lysozymes, and was accordingly renamed Ivy (*I*nhibitor of *v*ertebrate l*y*sozyme). Using Ivy-deficient and Ivy-overexpressing *E. coli* strains, we demonstrated that Ivy contributes to lysozyme resistance of *E. coli* when the bacteria are simultaneously challenged with lactoferrin or with high hydrostatic pressure to permeabilize their outer membrane [11], and these findings fed speculations about a possible role for lysozyme inhibitors in bacterial interactions with vertebrate hosts. Pleading against such a role in a wide range of bacteria is the limited distribution of Ivy homologs (only in a few proteobacterial species) and in particular their apparent absence in the majority of gram-negative pathogens.

However, until now no dedicated function-based screenings for lysozyme inhibitors in bacteria have been reported, and thus the existence of bacterial lysozyme inhibitors different from Ivy can not be excluded. This possibility is supported by our recent observation of lysozyme inhibitory activity in crude cell extracts of *Salmonella* Typhimurium and *S.* Enteritidis which do not contain an *ivy* homolog in their genome ([12] and unpublished observation). In the current paper, we report the identification of this component as a novel type of periplasmic proteinaceous lysozyme inhibitor unrelated to Ivy and we demonstrate that this inhibitor contributes to lysozyme resistance in *S.* Enteritidis. Furthermore, two other members of the large but cryptic family of proteins with which this novel inhibitor shares a common structural motif are demonstrated to inhibit lysozyme, supporting the functional annotation of this protein family as bacterial lysozyme inhibitors.

## Results

### Isolation and identification of a HEWL-inhibitor from *S.* Enteritidis

**Figure 1:**
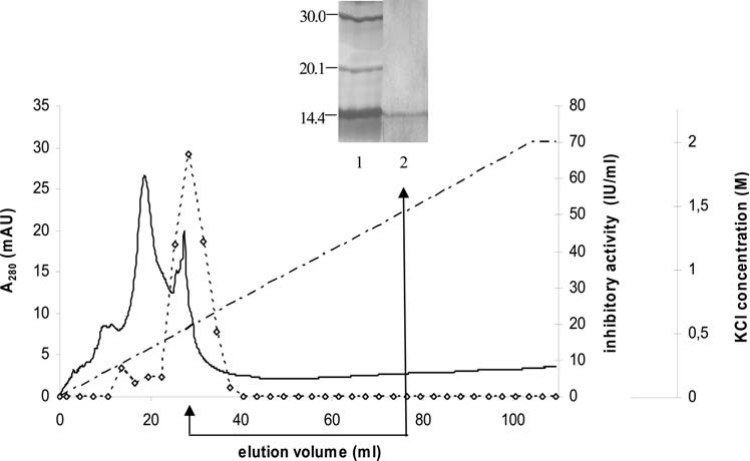
Purification of PliC from *S.* Enteritidis periplasmic extract by HEWL-affinity chromatography. Protein concentration in eluate was monitored by absorption at 280 nm [A_280_] and expressed in milli absorption units [mAU] (—), inhibitory activity of fractions against HEWL was monitored by inhibitor assay (-◊-). Elution was done with a gradient of 0–2.0 M KCl in 0.1 M Tris, pH 12.0 (·−·−·). Photograph in inset shows SDS-PAGE gel with molecular weight markers (lane 1, and indices in kDa at the left) and a concentrated fraction containing 0.163 mg/ml protein (without BSA addition) and an inhibitory activity of 323 IU/ml, corresponding to a specific activity of 1982 U/mg (lane 2).

In previous work we tested the sensitivity of cell walls of different gram-negative bacteria against several lysozymes [12]. To remove the outer membranes from these cells and make their cell walls accessible to lysozyme, we applied an extraction with chloroform-saturated buffer. A side observation in this work was that this procedure also allowed efficient extraction of the periplasmic lysozyme inhibitor Ivy from *E. coli* cells since extracts from the wildtype strain showed inhibitory activity against HEWL, while those from the Ivy^−^ strain did not. Interestingly, extracts from *S.* Typhimurium also showed HEWL inhibition, although *S.* Typhimurium does not contain an Ivy homolog, nor do any of the other *Salmonella* serotypes from which a genome sequence is available. This observation was extended to extracts of *S.* Enteritidis (data not shown). Since we previously purified Ivy by a single HEWL affinity chromatography step to more than 95% purity starting from a periplasmic extract of *E. coli* overexpressing Ivy from a plasmid [13], we used the same approach and the same matrix (HEWL coupled to N-hydroxysuccinimide-activated Sepharose 4 Fast Flow resin) to isolate the putative lysozyme inhibitor from wildtype *S.* Enteritidis. When the periplasmic extract obtained from *S.* Enteritidis (inhibitory activity of 11.6 IU/ml) was passed over the affinity column, the flow-through fraction did no longer show HEWL inhibitory activity. The elution of the bound proteins, with their corresponding inhibitory activity, is shown in Figure 1. Two peaks of 27 and 20 milli absorption units were detected at elution volumes of respectively 19 ml and 27 ml, the latter coinciding with a single peak of HEWL inhibitory activity (67 IU/ml). SDS-PAGE analysis of this active fraction showed only a single band after Coomassie or silver staining (Figure 1). Material recovered from a Coomassie band was subjected to trypsin digestion and tandem mass spectrometry analysis allowing to identify with high confidence peptides (MASGANYEAIDK, MASGANYEAIDKNYTYK, TAELVEGDDK and TAELVEGDDKPVLSNCSLAN) corresponding to fragments of the predicted product of the SEN1802 open reading frame in the genome sequence of *S.* Enteritidis PT4 (Wellcome Trust Sanger Institute, Cambridge UK; http://www.sanger.ac.uk/). A SEN1802 homolog is present in *S.* Typhimurium LT2 and all other sequenced *Salmonella* genomes (National Centre for Biotechnology Information; http://www.ncbi.nlm.nih.gov/). The function of this gene product is unknown but it carries a predicted N-terminal signal peptide of 24 amino acids for Sec dependent transport to the periplasm. This prediction is in good agreement with our isolation of the protein from the periplasmic cell fraction and with its supposed activity as a lysozyme inhibitor. SEN1802 has two cysteines in its amino acid sequence for possible disulfide bridge formation, a calculated pI of 4.76 and a predicted molecular weight of 9981 Da (for 90 amino acid residues) after cleavage of the signal peptide. This is less than our molecular weight estimation from gel migration (14.4 kDa), but such a deviation is not uncommon for acidic proteins and has been ascribed to poor binding of SDS [14]. Because of its HEWL inhibitory activity, we named the protein as PliC (*p*eriplasmic *l*ysozyme *i*nhibitor of *c*-type lysozyme).

### Overexpression and knock-out of *pliC* in *S.* Enteritidis

**Figure 2:**
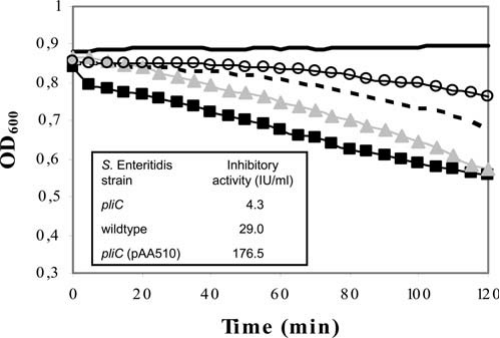
In vitro HEWL inhibitory activity of PliC from *S.* Enteritidis. Lysis (expressed as OD_600_ against time) of *M. lysodeikticus* cell suspension by 6.6 U/ml HEWL in the absence (-▪-) and presence of periplasmic protein extracts of *S.* Enteritidis *pliC* (1∶2 diluted; -▴-), *S.* Enteritidis (1∶10 diluted; —) and *S.* Enteritidis *pliC* (pAA510) (1∶40 diluted; -o-). Lysozyme inhibitory activity (IU/ml) of undiluted extracts is shown in the table in inset. The control sample (—) consisted of phosphate buffer instead of lysozyme solution added to *M. lysodeikticus*.

To investigate the function of PliC in *S.* Enteritidis, a PliC knock-out (*S.* Enteritidis *pliC*) and PliC overexpression strain (*S.* Enteritidis *pliC* (pAA510)) were constructed. The level of PliC production by these strains in comparison to the wildtype strain was evaluated by analyzing the lysozyme inhibitory activity of crude periplasmic protein extracts (Figure 2). Knock-out of PliC resulted in a strong reduction of inhibitory activity in extracts of *S.* Enteritidis *pliC* (4.3 IU/ml) compared to wildtype extracts (29.0 IU/ml). Since the open reading frame downstream of *pliC* has an opposite orientation, this loss of inhibitory activity cannot be due to a polar effect of the knock-out. Introduction of the pAA510 plasmid in *S.* Enteritidis *pliC* rescued lysozyme inhibitory activity (176.5 IU/ml when grown in the presence of 0.2% arabinose to induce the cloned *pliC* gene. These results confirm that the lysozyme inhibitory activity in the periplasmic extracts can be ascribed to the PliC protein. It should be remarked that the inhibitory activity of the wildtype extract in this experiment was higher than in the extract used for chromatographical purification (29.0 IU/ml versus 11.6 IU/ml). This is due to variability of yield between different osmotic shock treatments (data not shown). However, the yields of samples that were simultaneously processed in a single osmotic shock treatment were reproducible for a particular strain.

### PliC protects *S.* Enteritidis against HEWL in the presence of lactoferrin

**Figure 3:**
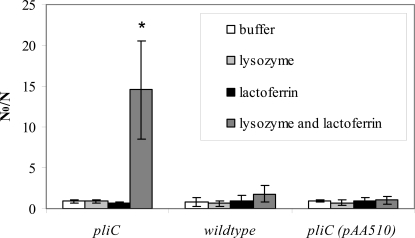
Sensitivity of *S.* Enteritidis strains to lysozyme in the presence of lactoferrin. Inactivation (N_o_/N) of *S.* Enteritidis *pliC*, *S.* Enteritidis and *S.* Enteritidis *pliC* (pAA510) after 24 h of incubation with 10 mM Tris-HCl pH 7.0 (□), 100 µg/ml lysozyme (▪), 3.0 mg/ml lactoferrin (▪) and lysozyme and lactoferrin together (▪). Mean values ± standard deviations (error bars) are shown (n = 4). Lysozyme treatments resulting in significant differences (p<0.01) compared to the same treatments without lysozyme are marked with an asterisk.

Suspensions of late exponential phase wildtype, *pliC* knock-out and *pliC* overexpression cells induced with arabinose were treated with 3.0 mg/ml lactoferrin, 100 µg/ml lysozyme, or a combination of both, and survivors were enumerated after 24 h (Figure 3). Most cells survived these treatments very well (inactivation levels not exceeding twofold), except for *S.* Enteritidis *pliC* cells in the presence of the lactoferrin - lysozyme mixture, which showed almost 15-fold inactivation. Lactoferrin is known to sensitize gram-negative bacteria to lysozyme and other antibacterial peptides by assisting their penetration through the outer membrane. Although the sensitizing action did not suffice to kill the wildtype *S.* Enteritidis under the conditions of our experiment, the fact that the *pliC* knock-out was sensitized demonstrates that natural levels of PliC were sufficient to protect *S.* Enteritidis cells against lysozyme.

### Distribution of PliC relatives

**Figure 4:**
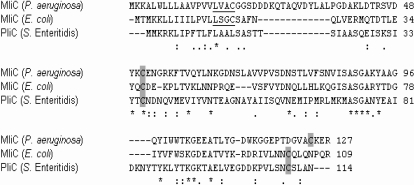
Amino acid sequence alignment of new HEWL inhibitors. Amino acid sequence alignment (http://www.ebi.ac.uk/clustalw/, [37]) of MliC from *P. aeruginosa* (  =  PA0867 from *P. aeruginosa* PA01), MliC from *E. coli* (  =  YdhA from *E. coli* MG1655) (both proteins containing the COG3895 domain) and PliC from *S.* Enteritidis (  =  SEN1802 from *S.* Enteritidis). Residues that are identical in all sequences in the alignment are marked with “*” in the bottom row, conserved and semi-conserved substitutions with “:” and “.” respectively. The lipobox of the lipoproteins is underlined, while cysteine residues of the mature protein are highlighted in grey.

An iterative search for sequences similar to the mature PliC protein using Psi-Blast [15] revealed besides the homologs in other *Salmonella* serotypes, similarity to proteins containing the conserved domain COG3895 (Clusters of Orthologous Groups, [16] http://www.ncbi.nlm.nih.gov/COG/). Proteins harboring this domain are widespread among members of the Proteobacteria, except the ε-Proteobacteria. Representatives are found in at least 52 different genera of the 155 completely sequenced genomes of all Proteobacteria available as to date (December 2007) and additionally occur in the Acidobacteria, Cyanobacteria and Bacteroides groups. The vast majority of COG3895 proteins are small proteins not containing other conserved protein domains and are predicted to be either periplasmic proteins (like PliC) or lipoproteins ([17], using the lipoprotein prediction tool available at http://www.mrc-lmb.cam.ac.uk/genomes/dolop/), but their function remains unknown. Also *E. coli* and *Pseudomonas aeruginosa*, which already have an active Ivy type lysozyme inhibitor [10],[18], encode a COG3895 protein, respectively YdhA and PA0867. These two proteins are predicted to be anchored to the periplasmic side of the outer membrane [19],[20]. Because of their homology with PliC of *Salmonella* and their cellular localization in the bacterial cell, these proteins were renamed as MliC (*m*embrane-bound *l*ysozyme *i*nhibitor of *c*-type lysozyme). This designation already anticipates on the functionality of these proteins as lysozyme inhibitors which will be demonstrated below. Although both bacteria, like *Salmonella*, belong to the γ-Proteobacteria, the two predicted MliC proteins share only 32% (over 53 amino acids) and 27% (over 65 amino acids) identity with PliC, and 38% identity (over 70 amino acids) with each other (Figure 4). Because of this relatively large distance and because a 3-D structure is available for MliC of *E. coli* (YdhA, [21]), MliC from *E. coli* and *P. aeruginosa* were chosen as representatives to further investigate the lysozyme inhibitory activity of the lipoprotein subgroup within the COG3895 group of proteins.

### In vitro HEWL-inhibitory activity of MliC proteins

**Figure 5:**
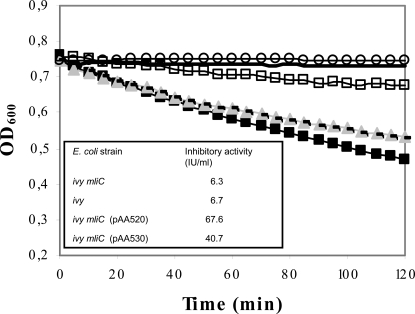
In vitro HEWL inhibitory activity of MliC from *P. aeruginosa* and *E. coli*. Lysis (expressed as OD_600_ against time) of *M. lysodeikticus* cell suspension by 6.6 U/ml HEWL in the absence (-▪-) and presence of membrane protein extracts of *E. coli ivy mliC* (1∶2 diluted; -▴-), *E. coli ivy* (1∶2 diluted; —), *E. coli ivy mliC* (pAA520) expressing MliC from *P. aeruginosa* (1∶10 diluted; -□-) and *E. coli ivy mliC* (pAA530) expressing MliC from *E. coli* (1∶5 diluted; -o-). Lysozyme inhibitory activity (IU/ml) of undiluted extracts is shown in the table in inset. The control sample (—) consisted of phosphate buffer instead of lysozyme solution added to *M. lysodeikticus*. The protein concentration of the undiluted membrane protein extracts from the different strains was the same (0.310 ± 0.045 mg/ml).

*mliC* from *P. aeruginosa* and *E. coli* were cloned under control of an arabinose inducible promoter (pAA520 and pAA530 respectively) in an *E. coli ivy mliC* background, to avoid interference from endogenous *E. coli* inhibitors. Lysozyme inhibitory activity was measured in the periplasmic extracts and membrane fractions of the overexpression strains after induction and compared to that of the control strain *E. coli ivy mliC* without overexpression plasmid. No significant differences in lysozyme inhibitory activity were found in the periplasmic protein extracts (data not shown). On the other hand, while only 6.3 IU/ml inhibitory activity was detected in the membrane fraction of *E. coli ivy mliC*, much higher levels of inhibitory activity were measured in the extracts upon induction of MliC expression from *P. aeruginosa* (67.6 IU/ml) or MliC from *E. coli* (40.7 IU/ml) (Figure 5). Therefore, we can conclude that both MliC of *P. aeruginosa* and MliC of *E. coli* are HEWL-inhibitors.

It can also be seen in Figure 5, that knock-out of *mliC* in *E. coli* had almost no influence on the level of inhibitory activity of the membrane extracts (6.7 versus 6.3 IU/ml, for an *ivy* and an *ivy mliC* strain respectively). This is in line with earlier reports that *mliC* (previously *ydhA*) transcripts of *E. coli* are not detected under normal laboratory growth conditions [22].

### Expression of the novel lysozyme inhibitors suppresses growth inhibition of *E. coli* by HEWL

**Figure 6:**
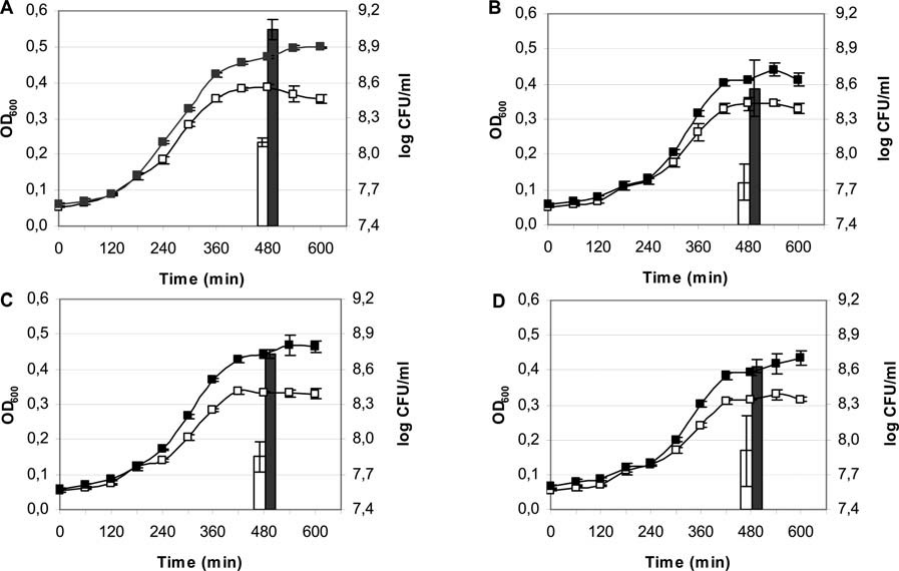
Influence of HEWL inhibitors on HEWL growth inhibition. Growth curves (OD_600_) of *E. coli tolA ivy mliC* harboring (A) pAA410 carrying Ivy from *E. coli*, (B) pAA510 carrying PliC from *S.* Enteritidis, (C) pAA520 carrying MliC from *P. aeruginosa*, and (D) pAA530 carrying MliC from *E. coli*, in the presence of 25 µg/ml HEWL and with (-▪-) or without (-□-) 0.02% arabinose. Bars represent viable cell numbers after 8 hours determined by plate count (log CFU/ml). Mean values ± standard deviations (error bars) are shown (n = 3).

To investigate the actual contribution of the inhibitors to bacterial HEWL resilience, *E. coli ivy mliC* was rendered sensitive to HEWL by introducing a *tolA* mutation that increases its outer membrane permeability. The resulting triple mutant was subsequently transformed with different plasmids that enable arabinose induced expression of either Ivy from *E. coli* (pAA410), PliC from *S.* Enteritidis (pAA510), MliC from *P. aeruginosa* (pAA520), and MliC from *E. coli* (pAA530). Next, we compared the growth inhibition by HEWL of these strains in the absence and in the presence of arabinose in the medium. At a HEWL concentration of 25 µg/ml, significant differences in optical density (OD_600_) and in plate counts (CFU/ml) of the cultures were observed upon induction of each inhibitor (Figure 6). Overexpression of Ivy, PliC of *S.* Enteritidis, MliC of *P. aeruginosa* or MliC from *E. coli* increased bacterial growth after 8 hours respectively 9, 7, 7 and 5-fold. A control construct (pAA100) containing the gene for green fluorescent protein (*gfp*) in the same vector and *E. coli* background, showed no significant differences in optical density or plate counts upon induction (data not shown). These results demonstrate that besides Ivy, also at least three members of the newly identified family of lysozyme inhibitors can effectively protect bacterial cells against lysozyme when expressed at appropriate levels.

## Discussion

In this work, we have identified a novel class of lysozyme inhibitors different from Ivy, the lysozyme inhibitor discovered earlier in *E. coli* [10]. These novel inhibitors belong to a large family of proteobacterial predicted periplasmic proteins or lipoproteins which share a common COG3895 structural motif with unknown function. We demonstrated lysozyme inhibitory activity for one periplasmic (PliC from *S.* Enteritidis), as well as for two lipoprotein members of this family (MliC from *P. aeruginosa* and from *E. coli*). Although no function had hitherto been assigned to any of the COG3895 proteins the 3-D solution structure of MliC from *E. coli* has been recently resolved, featuring an 8-stranded β-barrel, stabilized by a disulfide bond [21]. At the 3-D level, there is no resemblance with Ivy, which adopts a central β-sheet made of 5 antiparallel β-strands flanked on the convex side by two short helices and on the concave side by an amphipathic helix [18]. The Cys residues engaging in the disulfide bond in MliC from *E. coli* are conserved in both PliC from *S.* Enteritidis and MliC from *P. aeruginosa*, and in the majority of COG3895 proteins, suggesting that they may be important for preserving conformational stability.

The existence and possible function of lysozyme inhibitors in bacteria has not received much attention thus far. To our knowledge, a systematic screen for bacterial lysozyme inhibitors has not yet been conducted. This is surprising, given the important role of lysozymes in antibacterial defense in all major eukaryotic lineages, and the extensively documented existence of inhibitors of various other glycosyl hydrolases. Particularly plants produce a wide range of such inhibitors, for example against polygalacturonases, xylanases, α-amylases and β-glucanases, to thwart microbial attack. Therefore, the discovery in this work of a novel class of bacterial lysozyme inhibitors and the wide distribution of homologs of these inhibitors in the Proteobacteria may be indicative for their functional importance, for example in bacteria-host interactions. The location of the bacterial lysozyme inhibitors either in the periplasm (Ivy and PliC from *S.* Enteritidis), or anchored to the luminal face of the outer membrane (MliC from *E. coli* and *P. aeruginosa*) is also consistent with a role in protecting peptidoglycan from hydrolysis by exogenous lysozymes. In at least one instance more direct evidence for a role in host interaction exists. In *Salmonella* Typhi, expression of the *mliC* homolog was induced in cells residing within macrophages and knockout of *mliC* reduced macrophage survival [23]. Macrophages are known to produce a battery of antibacterial peptides including lysozyme and membrane permeabilizers, and hence the production of one or more lysozyme inhibitors by intracellular pathogens like *S.* Typhi makes sense from this point of view. The observed increased lysozyme sensitivity of an *S.* Enteritidis *pliC* knockout in the presence of 3.0 mg/ml of the outer membrane permeabilizing protein lactoferrin (Figure 3) provides a relevant indication in this context. Lactoferrin concentrations in this range occur in secretions like tears, airway mucus or colostrum [24],[25],[26]. Moreover, Ivy and all three new HEWL-inhibitors identified in this study suppressed growth inhibition by HEWL when overexpressed in an *E. coli* MG1655 *tolA ivy mliC* strain (Figure 6).

The genomic context of the newly identified lysozyme inhibitor genes also provides some interesting clues about their possible function. Immediately upstream of *pliC* of *S.* Typhimurium are the genes *pagC*, *pagD* and *msgA*, which play a role in macrophage survival of *S.* Typhimurium. Furthermore, transcriptome analysis has revealed that expression of *pliC* is controlled by SlyA, the same transcriptional activator that controls expression of *pagC* and *pagD* and that is necessary for virulence [27]. Based on its low GC content, the region encompassing *pagC* and a number of its immediate upstream genes was suggested to be acquired by lateral gene transfer, as is often the case for virulence genes [28]. The *pliC* gene, which is immediately downstream of *pagC*, also has a markedly lower GC content (42.0%) than the average of the LT2 chromosome (52.2%), and thus probably is an integral part of this acquired genome fragment. Interestingly, the *mliC* gene is located downstream of *slyA* in all sequenced *Salmonella* strains. Furthermore, both in *E. coli* and in *Salmonella*, *mliC* or its homolog are adjacent to *ydhH*, an open reading frame recently renamed to *anmK* because it encodes an anhydro N-acetyl muramic acid kinase involved in recycling of murein [29]. This allows speculation on a possible role of MliC in murein recycling, for example by controlling excessive hydrolysis of the murein backbone by lytic transglycosylases. However, at present we do not know whether the latter enzymes are inhibited by MliC or any of the other COG3895 proteins.

C-type lysozymes (e.g. HEWL or human lysozyme) are the major lysozymes produced by most vertebrates. In addition, all vertebrates have genes encoding g-type lysozyme. While the importance of the latter is not clear in man, it is the dominant type in some birds and it also occurs in fish species. A third type of lysozyme, called i-type, is characteristic for invertebrate animals such as arthropods, molluscs, nematodes etc. [30]. Neither PliC from *S.* Enteritidis, nor MliC from *E. coli* or *P. aeruginosa* have inhibitory activity against g-type lysozyme from goose egg white (data not shown). Ivy, in contrast, is active against goose egg white lysozyme [13] but not against g-type lysozyme from the urochordate *Oikopleura dioica* and i-type lysozyme from the scallop *Chlamys islandica* [31]. Given the existence and widespread occurrence of two types of c-type-specific lysozyme inhibitors in Proteobacteria, we anticipate that additional inhibitor classes specific against other types of lysozymes are also likely to be produced in bacteria. Screening of crude periplasmic extracts of a diverse range of bacteria for inhibitory activity against these g- and i-type lysozymes seems to corroborate this assumption (unpublished results), but definitive confirmation will have to await isolation and identification of the putative inhibitors.

The possible effect of bacterial lysozyme inhibitors in bacterial pathogenesis may even extend beyond neutralizing the direct antibacterial effect of lysozyme. Peptidoglycan has recently emerged as a powerful effector of the innate immune system through interaction with specific host receptors. The actual elicitor molecules are specific muropeptide fragments derived from peptidoglycan by bacterial and/or host lytic enzymes [32],[33]. This system of pattern recognition is believed to allow the host to distinguish pathogenic from non-pathogenic bacteria and to maintain its immune functions at an appropriate level. Malfunctioning of this system has been linked to chronic immune-related diseases such as inflammatory bowel disease and Crohn's disease. By interfering with the fragmentation of peptidoglycan by host lysozymes, bacterial lysozyme inhibitors can be anticipated to influence this system, and thus to play a potential role in these immune related pathologies. Provided that their role in bacterial pathogenesis can be further substantiated, bacterial lysozyme inhibitors may constitute an attractive new target for the development of anti-inflammatory and/or immunomodulating drugs.

In conclusion, we have identified a novel family of bacterial lysozyme inhibitors that contribute to bacterial lysozyme resistance and that have widespread homologs in gram-negative bacteria. Further study of these inhibitors will not only improve our understanding of bacteria-host interactions, lysozyme inhibitors may also turn out to be interesting novel targets for drug development.

## Materials and Methods

### Bacterial strains, plasmids, and culture conditions

Bacterial strains and plasmids used in this study are listed in Table 1. Construction of mutants and plasmids is discussed in Text S1. Where appropriate, plasmids were transformed to bacteria by electroporation.

**Table 1:** Bacterial strains and plasmids used in this study.

	Characteristics	Reference or source
Strains		
*Salmonella* Enteritidis		
wildtype	ATCC 13076	ATCC
*pliC* (*)	ATCC 13076 *pliC*::Cm	This study
*Escherichia coli*		
wildtype	MG1655	[38]
*mliC*(**)	MG1655 *mliC*::Kn (FB20404)	[39]
*ivy*	MG1655 *ivy*::Cm	This study
*ivy mliC*	MG1655 *ivy*::Cm *mliC*::Kn	This study
*tolA ivy mliC*	MG1655 Δ*tolA ivy*::Cm *mliC*::Kn	This study
*Pseudomonas aeruginosa*		
wildtype	PAO1	[40]
Plasmids		
pAA100	*gfp* gene under P*_BAD_* control, pFPV25 backbone, Ap^R^	[41]
pAA410	*ivy* gene of *E. coli* under P*_BAD_* control, pFPV25 backbone, Ap^R^	[11]
pAA510	*pliC* gene of *S.* Enteritidis under P*_BAD_* control, pFPV25 backbone, Ap^R^	This study
pAA520	*mliC* gene of *P. aeruginosa* (***) under P*_BAD_* control, pFPV25 backbone, Ap^R^	This study
pAA530	*mliC* gene of *E. coli* under P*_BAD_* control, pFPV25 backbone, Ap^R^	This study

*: *pliC*  =  SEN1802 from *S.* Enteritidis PT4 genome (www.sanger.ac.uk).

**: *mliC* of *E. coli*  =  *ydhA* from *E. coli* MG1655 genome (www.ncbi.nlm.nih.gov).

***: *mliC* of *P. aeruginosa*  =  PA0867 from *P. aeruginosa* PAO1 genome (www.ncbi.nlm.nih.gov).

All strains were originally cultured on Luria Bertani (LB; 10 g/l trypton, 5 g/l yeast extract, 5 g/l NaCl) agar plates and incubated at 37°C for 21 h. Overnight broth cultures were obtained by inoculating a single colony into LB broth containing appropriate antibiotics and incubating at 37°C for 21 h with aeration. Antibiotics (Sigma-Aldrich, Bornem, Belgium) were added when necessary to obtain the following final concentrations: 100 µg/ml ampicillin, 50 µg/ml kanamycin or 20 µg/ml chloramphenicol.

### Purification of HEWL-inhibiting proteins

For the purification of PliC, 500 ml cultures of *S.* Enteritidis ATCC 13076 were grown on a rotary shaker to stationary phase (21 h, shaking at 200 rpm) in LB at 37°C. Periplasmic cell extracts were then prepared by a gentle cold osmotic shock procedure as described earlier [13], and stored at −20°C until further analysis. Lysozyme binding inhibitors were isolated from this periplasmic cell fraction on an ÄKTA-FPLC platform (Amersham Pharmacia Biotech, Upsalla, Sweden) by affinity chromatography using immobilized HEWL as a ligand as described earlier for the Ivy protein [13], except that 100 ml of crude extract was loaded rather than 25 ml, and fractions of 5.0 ml rather than 2.0 ml were collected. The fractions were collected in tubes containing 300 µl of 1.0 M Tris-HCl pH 8.0 to neutralize the high pH of the elution buffer (pH 12.0), and bovine serum albumin (BSA, Sigma-Aldrich) was added to a final concentration of 0.5 mg/ml to stabilize the purified protein unless the samples were used for SDS-polyacrylamide gel electrophoresis (SDS-PAGE). Fractions were then desalted by dialysis against 10 mM potassium phosphate buffer pH 7.0 (12 kDa cut off, Sigma-Aldrich) and stored at −20°C until further use.

After purification, protein purity was checked with SDS-PAGE, conducted according to [34] with a 15% separating gel and a 4% stacking gel. Samples were prepared by boiling for 3 min in the presence of 1% ß-mercaptoethanol and 1% SDS. Gels were stained with Coomassie blue R 250 (Sigma-Aldrich), and, if higher sensitivity was desired, destained and subsequently silver-stained following the procedure of [35].

For the isolation of MliC of *P. aeruginosa* or *E. coli*, cultures of *E. coli ivy mliC* harboring plasmid pAA520 or pAA530 respectively, were grown overnight at 37°C in LB with ampicillin (100 µg/ml, Sigma-Aldrich), diluted 1/100 in fresh LB without antibiotics, induced with 0.2% (w/v) L−(+)-arabinose after 4 hours of growth, and further incubated at 37°C until stationary phase. Portions of 200 ml were subsequently harvested, resuspended in 10 ml 10 mM Tris-HCl pH 8 and lysed by three cycles of freezing and thawing followed by sonication (3×3 min, amplitude 40%, pulses 5 s on/5 s off). These suspensions were centrifuged for 1 hour at 100.000×*g* (4°C). The resulting pellet was resuspended in 10 ml 10 mM Tris-HCl buffer (pH 6.8) supplemented with 1.0 M NaCl, and sedimented again as described above. The membrane-bound proteins were then extracted using 2% Triton X-100 in a 10 mM Tris-HCl buffer (pH 6.8) supplemented with 10 mM MgCl_2_ and 150 mM NaCl and separated from insoluble material by centrifugation (1 hour at 100.000×*g*, 4°C).

### Protein identification by mass spectrometry

Active fractions containing the purified inhibitor protein were lyophilized, redissolved and subjected to SDS-PAGE and Coomassie staining. A gel fragment from the band corresponding to the inhibitor was trypsin-digested according to the method of [36], and the digests were then analyzed by electrospray tandem mass spectrometry on a LCQ Classic (ThermoFinnigan, San Jose, California) ion trap mass spectrometer equipped with a nano-liquid chromatography column switching system and a nanoelectrospray device. Tandem mass spectrometry data were searched using MASCOT (Matrix Sciences, London, U.K.) and SEQUEST (ThermoFinnigan) against the GenBank non-redundant protein database.

### Determination of HEWL inhibitory activity

Freeze-dried *M. lysodeikticus* ATCC4698 cells (Sigma-Aldrich) were resuspended at 0.5 mg/ml either in appropriate dilutions of the bacterial crude extracts, purified column fractions or in potassium phosphate buffer (10 mM, pH 7.0) with 0.5 mg/ml Bovine Serum Albumine (BSA) for the controls. Thirty µl of 66 U/ml HEWL (Hen Egg White Lysozyme; Fluka, 66000 U/mg protein) in potassium phosphate buffer (10 mM, pH 7.0) was then added to 270 µl of these suspensions and cell lysis was followed during 2 h at 25°C as the decrease in optical density (OD_600_) using a Bioscreen C Microbiology Reader (Labsystems Oy, Helsinki, Finland). In the absence of inhibitor, this procedure resulted in a linear OD_600_ decrease of 0.27 ± 0.04 over 2 h. The percentage inhibition (I) for each column fraction was calculated as:
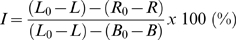
with L_0_ − L, R_0_ − R and B_0_ − B representing the OD_600_ decrease over a period of 2 h of the *M. lysodeikticus* suspensions respectively in the presence of lysozyme but with buffer instead of a bacterial extract/column fraction, in the presence of the bacterial extract/column fraction and lysozyme, and in the presence of the bacterial extract/column fraction but with buffer instead of lysozyme. Inhibitory activity was expressed in inhibitory units, with one unit being the amount of inhibitor that is needed to decrease the lysozyme activity by 50% under the above assay conditions.

### Sensitivity of *S.* Enteritidis to lysozyme in the presence of lactoferrin

*S.* Enteritidis, *S.* Enteritidis *pliC* and *S.* Enteritidis *pliC* (pAA510) cultures were grown overnight in LB with ampicillin and/or chloramphenicol when appropriate, diluted 1/100 in fresh LB without antibiotics, induced with 0.2% (w/v) L−(+)-arabinose (Fluka, Buchs, Switzerland), and incubated further. Arabinose served only to induce *pliC* expression from plasmid pAA510, but was also added to cultures of strains not carrying this plasmid to ensure identical culture conditions for all strains in the experiment. At an optical density (OD_600_) of 0.6 (5.10^8^ ± 1.10^8^ CFU/ml), cells were harvested by centrifugation (3800×*g*, 5 min) and subsequently resuspended in the same volume of Tris-HCl buffer (10 mM; pH 7.0) without and with lactoferrin (gift from Morinaga Milk Industries, Kanagawa, Japan; 3.0 mg/ml final concentration) and/or HEWL (Fluka, 66000 U/mg protein; 100 µg/ml final concentration). Samples were serially diluted in sterile Tris-HCl buffer (10 mM; pH 7.0) at the beginning and after 24 hours of treatment, and plated on LB agar plates to determine the degree of inactivation. Inactivation was expressed as a viability reduction factor, N_o_/N, with N_o_ and N respectively the colony counts at the beginning and after 24 hours of treatment.

### Lysozyme growth inhibition in vivo

Precultures of *E. coli* MG1655 *tolA ivy mliC* harboring plasmid pAA410, pAA510, pAA520, or pAA530 were grown overnight in LB broth containing ampicillin, kanamycin and chloramphenicol. Subsequently, cultures were diluted (1/100) in duplicate in fresh LB containing ampicillin, and after three hours of growth (exponential phase), either H_2_O or 0.02% L−(+)-arabinose was added, resulting in control and induced precultures respectively. These cultures were further grown to stationary phase to allow inhibitor expression. Subsequently, test tubes containing 4 ml LB with ampicillin, and either water or 0.02% L−(+)-arabinose and 25 µg/ml HEWL were inoculated (1/100) with the control and induced *E. coli* precultures respectively. These cultures were grown at 37°C during 10 hours. Each hour the OD_600_ was determined using a Multiscan RC (Thermo Scientific, Zellik, Belgium). After 8 hours the viable cell number was enumerated by plating on LB agar.

### List of geneID numbers

From *E. coli* MG1655: *ivy* (before *ykfE*): 946530 (Gene Entrez), *mliC* (before *ydhA*): 946811 (Gene Entrez), *tolA*: 946625 (Gene Entrez); From *P. aeruginosa*: *mliC* (before PA0867): 882238 (Gene Entrez); From *Salmonella* Enteritidis: *pliC*: SEN1802 (http://www.sanger.ac.uk/).

## Supporting Information

Text S1 The construction of the *S.* Enteritidis *pliC* knock-out mutant, the *E. coli ivy mliC* mutant, the *E. coli tolA ivy mliC* mutant and the construction of the plasmids pAA510, pAA520 and pAA530 is described.
(0.04 MB DOC)
