# Supplementary material for: A New Family of Lysozyme Inhibitors Contributing to Lysozyme Tolerance in Gram-Negative Bacteria
Source: PLoS Pathog. 2008 Mar 7;4(3):e1000019. doi: 10.1371/journal.ppat.1000019 (PMC2267010; doi:10.1371/journal.ppat.1000019)
Supplement: Text S1 — The construction of the S. Enteritidis pliC knock-out mutant, the E. coli ivy mliC mutant, the E. coli tolA ivy mliC mutant and the construction of the plasmids pAA510, pAA520 and pAA530 is described. (0.04 MB DOC) [file ppat.1000019.s001.doc]

Text S1

**1) Construction of *S.* Enteritidis *pliC* knock-out mutant:**

The *S.* Enteritidis *pliC* knock-out mutant was constructed using the standard one step inactivation protocol of [1]. A chloramphenicol resistance gene was amplified using Platinum *pfu* polymerase (Invitrogen, Merelbeke, Belgium) from the template plasmid pKD 3, using mutagenesis primers 5’-*aaaggatactaatgatgaaacgtaaattgatcccatttaccctct*gtgtaggctggagctgcttc-3’ and 5’-*acagttactgagaacaggtttatcatcaccttccaccagttcagc*catatgaatatcctcctta-3’. These primers contain a 5’-end extension of 45 nucleotides (italic) corresponding to a part of the *pliC* sequence to allow cross-over of the resistance gene into the genomic *pliC* gene after transformation of the amplified fragment to *S.* Enteritidis. Recombination was promoted by inducing the λ-red genes provided on pKD46 in the recipient *S.* Enteritidis strain with 1 mM L-arabinose (Fluka, Buchs, Switzerland) prior to transformation. A chloramphenicol resistant transformant was isolated and grown at 42 °C to cure plasmid pKD46. After confirmation of the *pliC* gene disruption by PCR and the absence of lysozyme inhibition in crude extracts, the transformant was designated *S.* Enteritidis *pliC*.

**2) Construction of *E. coli* *ivy* *mliC* mutant:**

To create an *ivy*::Cm allele, the *ivy* gene of plasmid pAA410 [2] was knocked out with a chloramphenicol resistance cassette, using the random transposon mutagenesis strategy described by [3]. Briefly, *E. coli* MG1655 pAA410 was grown to stationary phase in TBMM (10 g/l tryptone, 5 g/l NaCl, 0.2% maltose, 10 mM MgSO4) and mutagenized with phage λNK1324 as delivery vehicle for a transposon based chloramphenicol resistance marker. The culture was then plated on LB agar with 30 µg/ml chloramphenicol and incubated overnight at 39°C. Several plates with > 1000 transformants were pooled and plasmid was extracted from the pool with the High Pure Plasmid Purification Kit (Roche Diagnostics Belgium, Vilvoorde, Belgium). The pooled plasmids were transformed to MG1655 and plated on LB with 30 µg/ml chloramphenicol to select for the plasmids containing a transposon insertion. After overnight incubation at 37°C ca. 50 clones were analyzed by PCR to check which one of them contained the transposon in the *ivy* gene of pAA410. This *ivy*::Cm allele was amplified by PCR and transformed to MG1655 *mliC* (previously MG1655 *ydhA;* FB20404, kindly provided by Frederick Blattner) containing an induced pKD46 plasmid. After selection on chloramphenicol and curing of pKD46, this resulted in strain MG1655 *ivy* *mliC*.

**3) Construction of *E. coli* *tolA ivy* *mliC* mutant:**

The *E. coli* GL113 Δ*tolA*::Kn was kindly donated by Miguel A. Valvano (Departments of Microbiology and Immunology, University of Western Ontario, London, Ontario N6A5C1, Canada) [4]. From this strain a Δ*tolA*::Kn fragment was generated by PCR and transformed to *E. coli* MG1655 containing the induced pKD46 plasmid [1]. A resulting Kn-resistant transformant was subsequently transformed with the pCP20 plasmid encoding the FLP recombinase [1], in order to remove the Kn-resistance gene, resulting in the *E. coli* MG1655 *tolA* mutant. *E. coli* MG1655 *tolA* was subsequently equipped with the *mliC*::Kn and *ivy*::Cm alleles by transduction with a P1*vir* lysate grown on the *E. coli* MG1655 *ivy*::Cm *mliC*::Kn double knock-out, leading to the *E. coli* MG1655 *tolA ivy* *mliC* triple knock-out mutant.

**4) Construction of pAA510, pAA520 and pAA530:**

A plasmid carrying the *E. coli ivy* gene under control of the arabinose-inducible P*_BAD_* promoter, pAA410, was constructed earlier [2]. From this plasmid the *ivy* gene was spliced with *Xba*I and *Hin*dIII (Roche Diagnostics Belgium) and replaced with the *S.* Enteritidis *pliC* gene, the *mliC* gene of *P. aeruginosa*, or the *mliC* gene of *E. coli.* To this end, these genes were obtained by PCR amplification with Platinum *pfu* DNA polymerase using primers 5’-tcagtctagaaggatactaatgatgaaacg-3’ and 5’-tcagaagcttacaggaaggttgaagtgg-3’ for *pliC* from *Salmonella*; 5’-actgtctagatttctcgttagagggatttccat-3’ and 5’-cgataagcttcacggtggaccaacgggctg-3’ for the *mliC* gene of *P. aeruginosa* and 5’–tgctaagcttctttacggattgtcagtg–3’ and 5’–ctcgtctagatattgccctccagaccag-3’ for the *mliC* gene of *E. coli*. Subsequently, these PCR-products were cut with *Xba*I and *Hin*dIII before ligation. The resulting constructs contain the *pliC* gene of *S.* Enteritidis, the *mliC* gene of *P. aeruginosa*, and the *mliC* gene of *E. coli* under control of the P*_BAD_* promoter and were designated pAA510, pAA520 and pAA530 respectively.

1. Datsenko KA, Wanner BL (2000) One step inactivation of chromosomal genes in *Escherichia coli* K-12 using PCR-products Proc Natl Acad Sci USA 97:6640-6645.
2. Deckers D, Masschalck B, Aertsen A, Callewaert L, Van Tiggelen CGM et al. (2004) Periplasmic lysozyme inhibitor contributes to lysozyme resistance in *Escherichia coli.*  Cell Mol Life Sci 61:1229-1237.
3. Kleckner N, Bender J, Gottesman S (1991) Uses of transposons with emphasis on Tn10. Methods Enzymol 29:139-180.
4. Vinés ED, Marolda CL, Balanchandran A, Valvano MA (2005) Defective O-Antigen Polymerization in *tolA* and *pal* Mutants of *Escherichia coli* in Response to Extracytoplasmic Stress. J Bacteriol 187(10):3359-3368.
